# Effects of Dietary Intake of Japanese Mushrooms on Visceral Fat Accumulation and Gut Microbiota in Mice

**DOI:** 10.3390/nu10050610

**Published:** 2018-05-14

**Authors:** Takamitsu Shimizu, Koichiro Mori, Kenji Ouchi, Mamoru Kushida, Tsuyoshi Tsuduki

**Affiliations:** 1 Mushroom Research Laboratory, Hokuto Corporation, Nagano 381-0008, Japan; koichiro.mori@hokto-kinoko.co.jp (K.M.); k-namba@hokto-kinoko.co.jp (K.O.); 2 Laboratory of Food and Biomolecular Science, Graduate School of Agriculture, Tohoku University, Sendai 980-0845, Japan; mamoru.kushida.q3@dc.tohoku.ac.jp (M.K.); tsudukit@m.tohoku.ac.jp (T.T.)

**Keywords:** Japanese mushroom, obesity, gut microbiota, dietary fiber, dietary habits

## Abstract

A lot of Japanese people are generally known for having a healthy diet, and consume a variety of mushrooms daily. Many studies have reported anti-obesity effects of mushrooms, but few have investigated the effects of consuming a variety of edible mushroom types together in realistic quantities. In this study, we investigated whether supplementation with a variety of mushroom types affects visceral fat accumulation and gut microbiota in mice. The most popular mushroom varieties in Japan were lyophilized and mixed according to their local production ratios. C57BL/6J mice were fed a normal diet, high-fat (HF) diet, HF with 0.5% mushroom mixture (equivalent to 100 g mushrooms/day in humans) or HF with 3% mushroom mixture (equivalent to 600 g mushrooms/day in humans) for 4 weeks. The mice were then sacrificed, and blood samples, tissue samples and feces were collected. Our results show that mushroom intake suppressed visceral fat accumulation and increased the relative abundance of some short chain fatty acid- and lactic acid-producing gut bacteria. These findings suggest that mushroom intake is an effective strategy for obesity prevention.

## 1. Introduction

Japanese cuisine tends to include a greater diversity of ingredients, such as seafood, vegetables, soybeans and mushrooms, than European or American cuisine. Mushrooms are a particularly common ingredient in the Japanese cuisine, and have been used in cooking since about the 17th century [1]. Japanese people eat about 15 g of various kinds of mushrooms per day, which accounts for 0.68% of their total food intake [2]. Moreover, mushroom consumption is increasing in Japan following improvements in cultivation techniques that have stabilized mushroom supplies, and reports by many studies of the health effects of mushrooms [3,4]. The production volume of Japanese mushrooms was recently estimated at about 440,000 tons per year, with *Flammulina velutipes* (Enokitake) produced in the largest quantities, followed by *Hypsizygus marmoreus* (Bunashimeji), *Lentinus edodes* (Shiitake), *Grifola frondosa* (Maitake) and *Pleurotus eryngii* (Eringi or King oyster mushroom) [5]. These mushrooms contain many nutritional components such as dietary fiber, vitamin B_1_, vitamin B_2_, niacin, vitamin B_6_, vitamin D and folic acid [3], and are reported to have anti-obesity effects [6,7,8,9], immunomodulatory effects [10], anti-tumor effects [11], anti-atherosclerotic effects [12] and anti-diabetes effects [13]. However, these studies do not take into account the quantity of mushrooms realistically consumed as part of the diet.

Obesity is caused by an imbalance between the intake and expenditure of energy [14]. Compared with the American diet, the Japanese diet decreases the expression of stress responsive genes and increases that of metabolism-related genes, and thus has an anti-obesity effect [15,16]. However, the recent increase in animal fat in Japanese diets has led to a rapid increase in Japanese patients with lifestyle-related diseases [17,18]. To prevent or reduce obesity, the imbalance between energy intake and expenditure needs to be addressed by improving dietary habits and/or increasing physical activity, though this is difficult to achieve in modern society. Anti-obesity drugs, as an alternative option, have side effects and are expensive [19,20]. Conversely, the habitual consumption of foods with anti-obesity effects may be a cost-effective and manageable way to suppress obesity.

Gut bacteria are involved in host energy regulation and influence disease states such as obesity and diabetes [21,22]. The anti-obesity effects of improvements in gut microbiota have attracted attention following reports of obese mice and humans having a different gut microbiota to that of non-obese mice and humans [21,23]. Habitual diet is a major factor modulating the composition of gut microbiota [24]. Gut bacteria, which have beneficial effects for the host, proliferate using dietary fiber and oligosaccharides [25]. Some types of gut bacteria can produce lactic acid and short-chain fatty acids (SCFAs) (acetic acid, propionic acid and butyric acid), improve the intestinal environment and regulate host energy metabolism [26,27,28]. Increasing the numbers of these beneficial gut bacteria is suggested to have an anti-obesity effect.

Many studies have reported anti-obesity effects of mushrooms, but few have investigated the effects of consuming a variety of edible mushroom types together in realistic quantities. In this study, we investigated whether supplementation of a high-fat diet with a variety of mushroom types affects visceral fat accumulation and gut microbiota in mice.

## 2. Materials and Methods

### 2.1. Preparation of Mushroom Mixture

Enokitake was obtained from Mashgarden Corp. (Miyagi, Japan). Bunashimeji, Shiitake, Maitake and Eringi were obtained from Hokuto Corp. (Nagano, Japan). The fresh fruiting bodies of the mushroom were boiled in water for 10 min, then the water and treated mushrooms were freeze-dried. The Japanese mushrooms export/import is very low, and domestic consumption of mushrooms is high in Japan [5]. For this reason, the Japanese mushrooms production and Japanese mushroom consumption are almost the same. The freeze-dried mushroom powders were mixed in accordance with the ratios of Japanese mushroom production, as reported by the Ministry of Agriculture, Forestry and Fisheries of Japan [5] (Table 1). The nutritional composition (protein, fat, carbohydrate, water-soluble dietary fiber, insoluble dietary fiber) of the mushroom mixture was measured at the Japan Functional Food Analysis and Research Center (Fukuoka, Japan) (Table 2).

### 2.2. Preparation of the Test Diet

We prepared test diets of 0% (high fat, HF), 0.5% (mushroom low, ML) or 3% (mushroom high, MH) mushroom mixture in a high fat diet (D12079B, Research Diets, New Brunswick, NJ, USA). According to the National Health and Nutrition Survey of Japan [2], Japanese people eat about 15 g of various of mushrooms per day, which accounts for 0.68% of their total food intake. Therefore, consumption of 100 g of fresh mushrooms per day would account for about 5% of the Japanese daily intake. The dry weight of our mushroom mixture is 10% of fresh mushrooms. Thus, intake of test diets containing 0.5% or 3% mushroom mixture was assumed to be equivalent to an intake of about 100 g or 600 g, respectively, of fresh mushrooms per day in humans. To match the macronutrient balance and energy content of the test diets, a mimic mushroom mixture using casein as protein, soybean oil as lipid, cornstarch as carbohydrate, water-soluble dietary fiber as pectin powder, insoluble dietary fiber as cellulose powder and other as cellulose powder (Table 2) was added to the HF and ML diets as shown in Table 3. The energy content of the test diets was 460 kcal per 100 g. Finally, a low-fat diet containing 391 kcal per 100 g (98121701, Research Diets) was used as a control diet (control, CO).

### 2.3. Animals

All animal procedures were performed in accordance with the Animal Experiment Guidelines of Tohoku University, and the animal protocol was approved by the Animal Use Committee at Tohoku University (Registration ID No. 2016Noudou-009). Four-week-old male C57BL/6J mice (mean body weight: 18 g) were obtained from SLC, Inc. (Shizuoka, Japan). Mice were housed for the duration of the study under a 12 h/12 h light/dark cycle in a temperature and humidity controlled room, and fed a standard rodent chow (CE-2, CLEA Japan, Inc., Tokyo, Japan) for 1 week to acclimate. At 5 weeks old, the mice were randomly divided into four dietary groups (*n* = 8 in each group, 4 mice per cage), with each group receiving either CO, HF, ML or MH (Table 3) for four weeks with free access to food and water. At the end of the 4-week period, mice were weighed and blood samples were collected following decapitation under isoflurane anesthesia. Brain, heart, lung, liver, spleen, pancreas, kidney, small intestine, large intestine and white adipose tissues were removed and weighed. Serum samples and organs were stored at −80 °C until use.

### 2.4. Histological Analysis

Perinephric adipose tissue and liver were fixed in 10% formalin and embedded in paraffin [29]. Vertical sections (4 µm) were cut, mounted on a glass slide, stained with hematoxylin and eosin, and observed using a microscope (BZ-9000; Keyence, Osaka, Japan). The mean adipocyte area was calculated.

### 2.5. Biochemical Analyses in Serum and Liver

The lipid composition of liver and serum samples was measured as described previously [30]. Triacylglycerol (TG) and total cholesterol (TC) in serum and liver, and phospholipid (PL), glucose, alanine aminotransferase (ALT) and aspartate transaminase (AST) in serum were measured using commercial enzyme kits (Wako Pure Chemical, Osaka, Japan). Insulin in serum was determined using an ELISA kit (Morinaga Institute of Biological Science, Kanagawa, Japan). Interleukin (IL)-6 in serum was determined using an ELISA kit (BD Biosciences, Franklin Lakes, NJ, USA). Adiponectin in serum was determined using an ELISA kit (Otsuka Pharmaceutical Co., Ltd., Tokyo, Japan). PL in liver was determined using the method described by Rouser [31]. To examine oxidative stress caused by the diet, we measured the levels of thiobarbituric acid active substances (TBARS) in serum and liver as described previously [32].

### 2.6. mRNA Expression Analysis

For real-time quantitative reverse transcriptase PCR, total RNA was isolated from perinephric adipose tissue using an RNeasy Mini Kit (Qiagen, Valencia, CA, USA) [33,34], eluted with 40 µL RNase-free water, and stored at −80 °C until use. To quantify gene expression levels, mRNA levels for adiponectin (*Adipoq*), fatty acid synthase (*Fas*), glucose-6-phosphate dehydrogenase X-linked (*G6pdx*), hormone-sensitive lipase (*Hsl*), malic enzyme (*Me*), peroxisome proliferator activated receptor gamma (*Pparγ*), sterol regulatory element binding factor 1 c (*Srebp-1c*), and beta-actin (*β-actin*) in perinephric adipose tissue were determined with a Thermal Cycler Dice Real Time System (Takara Bio Inc., Otsu, Japan). In brief, cDNA was made using Prime Script RT Master Mix (Takara Bio Inc.) from total RNA in perinephric adipose tissue. This cDNA was subjected to PCR amplification using SYBR Premix Ex TaqTM (Takara Bio Inc.) and gene-specific primers for *Adipoq*, *Fas*, *G6pdx*, *Hsl*, *PPARγ*, *Srebp-1c* and *β-actin* (Table 4). PCR amplification was performed with an activation step of 95 °C for 10 s, followed by 40 cycles of 95 °C for 5 s (denaturation) and 60 °C for 31 s (extension), and a dissociation stage of 95 °C for 15 s, 60 °C for 30 s and 95 °C for 15 s, for each gene. The *β-actin* content in test samples was used as the normalization factor.

### 2.7. Gut Microbiota Analysis

Feces were collected at 0 weeks (start of test period), 2 weeks and 4 weeks just after routine cage changing. Collected feces were pooled into a single-tube for each group, and stored at −80 °C until use. Total genome DNA was extracted from feces using a DNasey Powersoil kit (Qiagen) following the manufacturer’s instructions. The V3-V4 region of the *16S* rRNA gene was amplified using forward primer 341 F (5′-TCGTCGGCAGCGTCAGATGTGTATAAGAGACAGCCTACGGGNGGCWGCAG-3′) and reverse primer 806 R (5′-GTCTCGTGGGCTCGGAGATGTGTATAAGAGACAGGGACTACHVGGGTWTCTAAT-3′) (Sigma-Aldrich, St. Louis, MI, USA). PCR cycling conditions consisted of an initial denaturation of 1 min at 94 °C, and 28 cycles of 10 s at 98 °C, 15 s at 30 °C and 15 s at 68 °C. The presence of the amplified *16S* rRNA gene band was verified in an agarose gel. The PCR products were amplified in a second PCR employing Nextera XT Index primer (Illumina, Inc., San Diego, CA, USA). This PCR was run for 1 min at 94 °C, followed by 8 cycles of 10 s at 98 °C, 15 s at 60 °C and 15 s at 68 °C. Amplicon sequencing was performed on the Illumina MiSeq system (Illumina, San Diego, CA, USA).

### 2.8. Statistical Analysis

All statistical analyses were performed using KaleidaGraph (HULINKS Inc., Tokyo, Japan). Results are expressed as mean ± standard error. Data were analyzed by one-way ANOVA with a Dunnett test. A difference was considered to be significant at *p* < 0.05.

## 3. Results

### 3.1. Effects of Mushroom Intake on Growth Parameters

**Figure 1:**
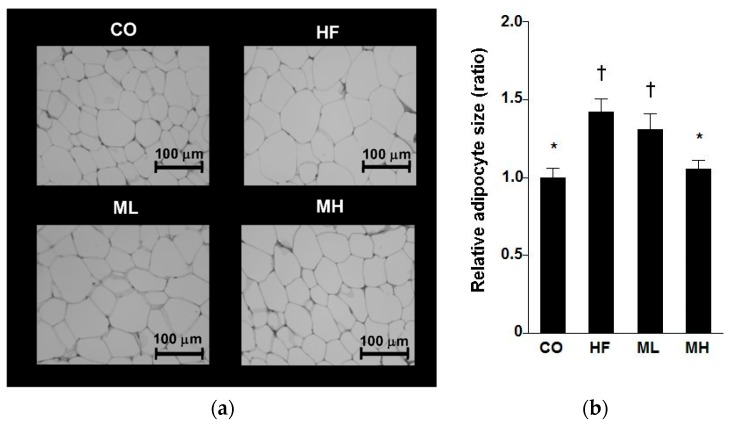
Effect of mushroom intake on white adipose tissue in mice. (**a**) Perinephric adipose tissue sections from mice of each group (hematoxylin and eosin, scale bar = 100 µm); (**b**) Average size of adipocytes in perinephric adipose tissue. Values are mean ± standard error, *n* = 8. CO: control diet, HF: high fat diet, ML: mushroom low (0.5%) diet, MH: mushroom high (3%) diet. ^†^: *p* < 0.05 (vs. CO group), *: *p* < 0.05 (vs. HF group).

There were no significant differences in body weight or weight gain between groups (Table 5). Food intake was significantly greater in the CO group than in the HF, ML or MH groups, but there were no significant differences among groups in energy intake. The ML and MH groups showed a significant decrease in heart weight compared with the CO group. But, there is no significant difference in measured value of the heart (data not shown). The weight of the large intestine in the ML group was significantly greater than that in the HF group. Mushroom intake decreased the weight of white adipose tissue in a dose-dependent manner compared with the HF group, and the MH group showed significant decrease in perinephric adipose tissue weight compared with the HF group. There were no other significant differences in organ weights among the groups. Because there was a significant difference in the perinephric adipose tissue weight, histological analysis was performed by staining perinephric adipose tissue with hematoxylin-eosin. The average size of adipocytes in the MH group was significantly smaller than that in the HF group (Figure 1). These results indicate that mushroom intake suppressed visceral fat accumulation.

### 3.2. Biochemical Parameters in Serum and Liver

**Figure 2:**
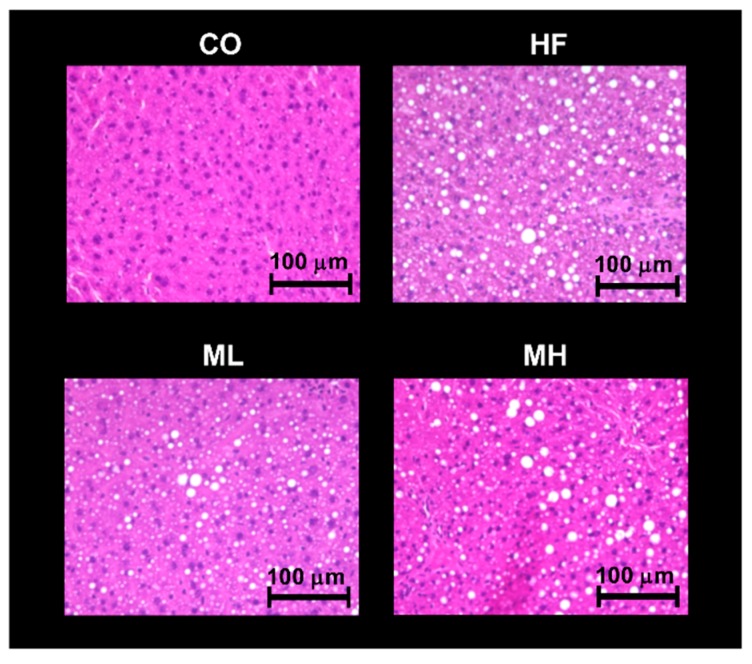
Effect of mushroom intake on liver histology in mice. Liver sections from mice of each group (hematoxylin and eosin, scale bar = 100 µm). CO: control diet, HF: high fat diet, ML: mushroom low (0.5%) diet, MH: mushroom high (3%) diet.

We investigated the effect of mushroom intake on serum lipid metabolism (TC, TG and PL), lipid peroxidation (TBARS), glucose metabolism (glucose and insulin), liver injury (ALT and AST) and inflammation (IL-6) (Table 6). Serum TC levels were significantly higher in the HF, ML and MH groups than the CO group, and significantly higher in the ML group than the HF group. Serum PL levels were significantly higher in the ML and MH groups than the CO group, and significantly higher in the ML group than the HF group. Serum TBARS levels were significantly lower in the HF, ML and MH groups than the CO group, and significantly higher in the ML group than the HF group. The serum level of adiponectin was significantly higher in the MH group than the CO or HF groups. There were no significant differences in serum TG, glucose, insulin, ALT, AST or IL-6 levels among groups. We then examined the effect of mushroom intake on liver TC, TG, PL and TBARS (Table 6). Liver TC levels were significantly higher in the HF, ML and MH groups than the CO group, and significantly higher in the ML group than the HF group. Mushroom intake increased liver TG levels in a dose-dependent manner compared with the HF group, and the TG level was significantly higher in the MH group than the HF group. Liver TBARS levels were significantly lower in the HF and MH groups than the CO group, and significantly higher in the ML group than the HF group. There were no significant differences in liver PL levels among groups. Since the TG level in the liver increased in a dose-dependent manner with mushroom intake, a histological analysis was performed by staining liver with hematoxylin-eosin to observe lipid droplets. An increase in the number of lipid droplets in the liver was observed in the HF group compared with the CO group, but there were no differences among the HF, ML and MH groups (Figure 2). These results suggest that mushroom intake did not significantly affect lipid or glucose metabolism, but increased serum adiponectin levels.

### 3.3. mRNA Expression in Perinephric Adipose Tissue

Mushroom intake decreased the weight of perinephric adipose tissue and suppressed adipocyte hypertrophy. To elucidate the mechanism underlying this effect, mRNA levels of lipid metabolism-related genes in perinephric adipose tissue were measured using real-time quantitative reverse transcriptase PCR. As shown in Table 7, the fatty acid synthesis-related genes *Fas*, *Me*, *G6pdx* and *Srebp-1c* showed different responses. *Me* expression was significantly decreased in the HF and ML groups compared with the CO group. *G6pdx* expression increased with mushroom intake in a dose-dependent manner compared with the HF group, and was significantly higher in the MH group than the CO or HF groups. *Srebp-1c* expression was significantly higher in the HF and ML groups than the CO group, and decreased with mushroom intake in a dose-dependent manner compared with HF group so that *Srebp-1c* expression was significantly lower in the MH group than the HF group. The expression of *Hsl*, a lipolysis-related gene, increased with mushroom intake in a dose-dependent manner, and was significantly higher in the MH group than the CO or HF groups. The expression of *Fas*, a gene related to the synthesis of fatty acids, showed no significant differences among groups. The expression of *Pparγ*, a cell differentiation-related gene, increased with mushroom intake in a dose-dependent manner, and was significantly higher in the MH group than the CO group. These results indicate that dietary mushroom intake suppressed fatty acid synthesis, promoted lipolysis and promoted differentiation and increase of adipocytes, thereby inhibiting fat accumulation in white adipose tissue. As the MH group had increased serum adiponectin levels (Table 6), we measured *Adipoq* expression levels in adipocytes to determine whether mushroom intake regulates adipocyte adiponectin production. The results showed that adipocyte *Adipoq* expression increased with mushroom intake in a dose-dependent manner, and was significantly increased the MH group compared with the CO and HF groups (Table 7). These results indicate that dietary mushroom intake promoted adiponectin production in adipocytes.

### 3.4. Effects of Mushroom Intake on Gut Microbiota

**Figure 3:**
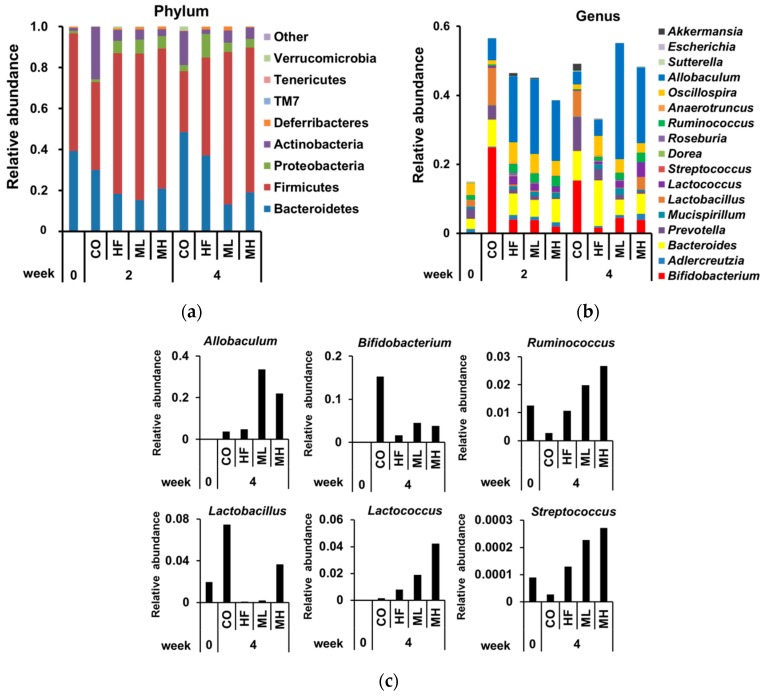
Modulation of gut microbiota by mushroom intake. Fecal microbiota composition of mice after 0 weeks, 2 weeks and 4 weeks on a control diet (CO), high fat diet (HF), mushroom low (0.5%) diet (ML), or mushroom high (3%) diet (MH). (**a**) Phylum level and (**b**) genus level taxonomic distributions of fecal microbial communities determined by next generation sequencing. (**c**) Relative abundance of short chain fatty acid-producing bacteria (*Allobaculum, Bifidobacterium* and *Ruminococcus*) and lactic acid-producing bacteria (*Lactobacillus*, *Lactococcus* and *Streptococcus*).

At the phylum level after 2 weeks, the relative abundance of Bacteroidetes and Actinobacteria was lower in the HF, ML and MH groups than the CO group, and the relative abundance of Firmicutes and Proteobacteria was higher in the HF, ML and MH groups than the CO group (Figure 3). There were no significant changes at the phylum level after 2 weeks among the HF, ML and MH groups. At the phylum level after 4 weeks, the relative abundance of Bacteroidetes was lower in the HF, ML and MH groups than the CO group, and lower in the ML and MH groups than the HF group. The relative abundance of Firmicutes was higher in the HF, ML and MH groups than the CO group, and higher in the ML and MH groups than the HF group. The relative abundance of Proteobacteria was higher in the HF group than the CO group, and lower in the ML and MH groups than the HF group. The relative abundance of Actinobacteria was lower in the HF group than the CO group, and higher in the ML and MH groups than the HF group. The relative abundance of Deferribacteres and Verrucomicrobia was lower in the MH group than the HF group. There were no significant differences in the relative abundance of Tenericutes, TM7 or “Other” among the HF, ML and MH groups. The relative abundance of each bacterial phylum is given in Table S1. We focused on the gut bacterial genera whose relative abundance in the MH group after 4 weeks was more than 2-fold or less than half that of the HF group after 4 weeks. At the genus level after 2 weeks, there were significant differences in the gut microbiota between the HF and CO groups, but not among the HF, ML and MH groups. At the genus level after 4 weeks, the gut microbiota of the ML and MH groups was significantly different to that of the HF group. The ML and MH groups showed a tendency towards increased SCFA-producing bacteria (*Allobaculum*, *Bifidobacterium* and *Ruminococcus*) and lactic acid-producing bacteria (*Lactobacillus, Lactococcus* and *Streptococcus*) compared with the HF group. The MH group tended to have increased *Adlercreutzia* and *Sutterella* and decreased *Bacteroides*, *Prevotella*, *Mucispirillum*, *Dorea*, *Roseburia*, *Anaerotruncus*, *Oscillospira*, *Escherichia* and *Akkermansia* compared with the HF group. The relative abundance of each bacterial genus is given in Table S2. These results suggest that the gut microbiota composition changed over time with mushroom intake, leading to an increase in some SCFA- and lactic acid-producing bacteria at 4 weeks.

## 4. Discussion

Our study shows that dietary intake of a variety of mushrooms is effective in preventing obesity by suppressing visceral fat accumulation. Similarly, many studies have reported that the dietary fiber contained in mushrooms has an anti-obesity effect [8,35,36]. Mushrooms are known to contain a large amount of dietary fiber [37], and the mushroom mixture used in this study contained 5.20% water-soluble dietary fiber and 30.5% insoluble dietary fiber (Table 2). The dietary fiber contained in mushrooms is glucan, lignin, pectin and chitin [38,39]. Although water-soluble dietary fiber has a weaker anti-obesity effect than insoluble dietary fiber [40], pectin, which is water-soluble, has the effect of suppressing feeding [41]. Furthermore, water-soluble dietary fibers have been shown to prevent diabetes [42], improve serum cholesterol and increase SCFA concentrations in the body [43]. Insoluble dietary fibers, such as insoluble glucan, lignin and chitin, increase the amount of feces because of their hygroscopic properties, and promote lipid adsorption and discharge [40]. We hypothesized that the dietary fiber component of the mushrooms was likewise involved in the prevention of fat accumulation in our study. Pectin and cellulose were added to the HF diet to match the amount of dietary fiber in the MH diet; nevertheless, the anti-obesity effect of the MH diet was greater than that of the HF group. Thus, we suggest that intake of different types of dietary fiber from a variety of mushrooms suppresses fat accumulation. Furthermore, dietary fiber increases the weight and length of colon [44]. We hypothesized that the dietary fiber of mushrooms involved in the increase in weight and length of large intestine in our study.

SREBP-1c enhances the gene expression of fatty acid synthesis factors such as FAS, inducing the conversion of glucose to fatty acids and triacylglycerols for storage [45,46]. There have been reports that mushroom components [47,48] and 25-hydroxyvitamin D [49] suppress SREBP-1c and FAS, but no studies have examined the effects of edible mushroom intake in mice. The results of this study show that mushroom intake decreased *Srebp-1c* gene expression in a dose-dependent manner compared with the HF group. Thus, we show that mushrooms inhibited the synthesis of fatty acids even when ingested as part of a diet. Moreover, our results show that mushroom intake increased the gene expression of *G6pdx*, which is involved in fatty acid synthesis. G6PDH produces nicotinamide adenine dinucleotide phosphate [50], which suppresses oxidation along with antioxidant enzymes [51]; thus, *G6pdx* activation may have an antioxidant effect.

In this study, mushroom intake increased the gene expression levels of *Pparγ* and *Adipoq*. PPARγ regulates the differentiation from pre-adipocyte to adipocyte [52]. Obesity occurs through an increase in the number and size of adipocytes with the excessive accumulation of triacylglycerols; therefore, a PPARγ-mediated increase in adipocytes enhances obesity. Moreover, high levels of PPARγ expression increase the number of small adipocytes and decrease number of large ones [53]. Small adipocytes reduce the production of inflammatory cytokines that promote metabolic syndrome, and increase the production of adiponectin that prevents metabolic syndrome [54]. We hypothesized that mushroom intake suppressed adipocyte hypertrophy through activation of PPARγ, because mushroom intake suppressed the enlargement of adipocytes and promoted adiponectin production in the present rodent study.

Mushroom intake also affected the gut microbiota of our HF diet-fed mice. Gut microbiota has been shown to affect the progression of obesity in the host. Obese mice exhibit high Firmicutes and low Bacteroidetes at the phylum level compared with normal mice [19,21]. Intake of dietary fiber decreases Firmicutes and increases Bacteroidetes [55]. In this study, despite its suppression of obesity, mushroom intake increased Firmicutes and decreased Bacteroidetes compared with the HF group. This was the result of an increase in the Firmicutes genera *Allobaculum*, *Lactobacillus, Lactococcus*, *Ruminococcus* and *Streptococcus*. Some Firmicutes gut bacteria grow using dietary fiber and produce lactic acid and SCFAs [56]. This increase in intestinal lactic acid results in the proliferation of SCFA-producing bacteria, prevention of intestinal inflammation and suppression of obesity [25]. In the present study, an increase in lactic acid-producing bacteria (*Lactobacillus, Lactococcus* and *Streptococcus*) and SCFA-producing bacteria (*Allobaculum, Bifidobacterium* and *Ruminococcus*) was observed with mushroom intake. This could be because these bacteria thrived on the dietary fiber from the mushrooms [28,57,58,59]. SCFAs produced by these bacteria promote energy metabolism in the host [26]. Thus, we hypothesized that mushroom intake increased SCFA concentrations in the body, and that these played a role in suppressing fat accumulation.

TG in the liver accumulates following absorption of dietary lipids from the small intestine or synthesis in the liver [60]. The chitin/chitosan contained in mushrooms has been shown to suppress accumulation of liver TG by suppressing the absorption of diet-derived lipid [61,62]. However, in the present study, the TG level in the liver increased with mushroom intake. One explanation for this is that mushroom intake promoted *Hsl* expression in adipose tissues, which induces the breakdown of adipocyte TG into free fatty acids that are then released into the blood. When not consumed as energy, free fatty acids reach the liver where they are resynthesized as TG [63]. Therefore, liver TG in the MH group may have been elevated because of increased free fatty acids following the promotion of TG decomposition in adipocytes. Notably, there were no significant differences in serum ALT or AST or liver TBARS between the HF group and the ML and MH groups. Thus, we suggest that the liver TG accumulation resulting from mushroom intake did not cause inflammation and oxidative stress. In addition, histological analysis showed that there were no significant differences in the lipid droplets in the liver between the HF and MH groups, indicating that mushroom intake did not adversely affect the liver. It is known that liver TG and lipid droplets are increased by Shiitake, among the edible mushrooms [64,65], possibly as a result of the eritadenine content of Shiitake [65,66]. Eritadenine inhibits S-adenosyl-L-homocysteine hydrolase activity in the liver and increases S-adenosyl-L-homocysteine concentration, suppressing phosphatidylcholine (PC) synthesis by methylation of phosphatidylamine (PE) and increasing PE concentration [66]. The PC/PE ratio affects cell membrane permeability of the liver [67]. Intake of Shiitake decreases the PC/PE ratio of the liver [65,66], which promotes fatty liver. Therefore, we suggest that the Shiitake contained in our test diet played a role in the increase in liver TG following mushroom intake. To compensate for this effect, it may be beneficial to eat foods that promote the metabolism of liver fat, such as the EPA and DHA contained in fish, at the same time as eating mushrooms [68]. Further studies are needed to elucidate the mechanisms underpinning the metabolic effects of mushroom intake.

## 5. Conclusions

Our results show that mushroom intake suppressed fat accumulation by inhibiting fatty acid synthesis and promoting lipolysis of visceral fat. Mushroom intake also suppressed adipocyte enlargement and promoted adiponectin production. Moreover, mushroom intake caused some lactic acid- and SCFA-producing bacteria to proliferate, possibly because of the dietary fiber content of the mushrooms. Thus, we speculate that mushroom intake increases the production of SCFAs, which promote energy metabolism in the host and play a role in suppressing fat accumulation. In conclusion, our findings suggest that mushroom intake is an effective strategy for the prevention of obesity.

## Figures and Tables

**Table 1:** Proportion of each mushroom species in the mushroom mixture.

Mushroom	Ratio (%)
*Flammulina velutipes*	33.0
*Hypsizygus marmoreus*	28.2
*Lentinu* *s* *edodes*	17.2
*Grifola frondosa*	12.0
*Pleurotus eryngii*	9.60

**Table 2:** Nutritional composition of the mushroom mixture and the mimic mushroom mixture.

Mushroom Mixture		Mimic Mushroom Mixture	
	(g/100 g)		(g/100 g)
Protein	21.6	Casein	21.6
Fat	1.60	Soybean oil	1.60
Carbohydrate	26.2	Cornstarch	26.2
Water-soluble dietary fiber	5.20	Pectin powder	5.20
Insoluble dietary fiber	30.5	Cellulose powder	30.5
Other	14.9	Cellulose powder	14.9
	(kcal/100 g)		(kcal/100 g)
Energy	216		216

**Table 3:** Composition of the test diets.

	CO	HF	ML	MH
Control diet	100%	0%	0%	0%
High-fat diet	0%	97%	97%	97%
Mushroom mixture	0%	0%	0.5%	3%
Mimic mushroom mixture	0%	3%	2.5%	0%
Energy (kcal/100 g)	391	460	460	460

CO: control diet, HF: high fat diet, ML: mushroom low (0.5%) diet, MH: mushroom high (3%) diet.

**Table 4:** Primer pairs used for the real-time quantitative PCR analysis.

Genbank ID	Gene Name	Primer Sequence (5′ to 3′)	
NM_009605	*Adipoq*	Forward	TGTTCCTCTTAATCCTGCCCA
		Reverse	CCAACCTGCACAAGTTCCCTT
NM_007393	*β-actin*	Forward	GAAATCGTGCGTGACATCAAAG
		Reverse	TGTAGTTTCATGGATGCCACAG
NM_007988	*Fas*	Forward	CCTGGATAGCATTCCGAACCTG
		Reverse	TCACAGCCTGGGGTCATCTTTGC
NM_008062	*G6pdx*	Forward	TGGGTCCACCACTGCCACTTTTG
		Reverse	ATTGGGCTGCACACGGATGACCA
NM_001039507	*Hsl*	Forward	TTCTCCAAAGCACCTAGCCAA
		Reverse	TGTGGAAAACTAAGGGCTTGTTG
M29546	*Me*	Forward	GAAAGAGGTGTTTGCCCATGA
		Reverse	AATTGCAGCAACTCCTATGAGG
NM_011146	*Pparγ*	Forward	TCGCTGATGCACTGCCTATG
		Reverse	GAGAGGTCCACAGAGCTGATT
NM_011480	*Srebp-1c*	Forward	GGAGACATCGCAAACAAGC
		Reverse	TGAGGTTCCAAAGCAGACTG

*Adipoq*: Adiponectin, *β-actin*: beta-actin, *Fas*: fatty acid synthase, *G6pdx*: glucose-6-phosphate dehydrogenase X-linked, *Hsl*: hormone-sensitive lipase, *Me*: malic enzyme, *Pparγ*: peroxisome proliferator activated receptor gamma, and *Srebp-1c*: sterol regulatory element binding factor 1 c.

**Table 5:** Body weights, food intake, tissue weights and large intestine lengths.

	CO	HF	ML	MH
Body weight (g)				
Final (g)	26.6 ± 0.5	27.0 ± 0.6	28.2 ± 0.5	28.0 ± 0.5
Gain (g)	6.50 ± 0.51	7.23 ± 0.74	8.32 ± 0.59	7.93 ± 0.63
Food intake (g/day)	3.70 ± 0.22 *	2.93 ± 0.10 ^†^	2.88 ± 0.11 ^†^	2.74 ± 0.09 ^†^
Energy intake (kcal/day)	14.5 ± 0.9	13.5 ± 0.5	13.3 ± 0.5	12.6 ± 0.4
Tissue weight (g/100 g Body weight)				
Brain	1.72 ± 0.05	1.68 ± 0.03	1.69 ± 0.03	1.66 ± 0.04
Heart	0.519 ± 0.018	0.490 ± 0.013	0.454 ± 0.012 ^†^	0.458 ± 0.013 ^†^
Lung	1.22 ± 0.15	0.941 ± 0.07	1.03 ± 0.10	0.887 ± 0.10
Liver	3.96 ± 0.15	3.88 ± 0.07	3.81 ± 0.06	3.68 ± 0.05
Pancreas	0.703 ± 0.082	0.752 ± 0.043	0.826 ± 0.045	0.742 ± 0.043
Spleen	0.280 ± 0.022	0.290 ± 0.018	0.297 ± 0.012	0.252 ± 0.016
Kidney	1.17 ± 0.02	1.14 ± 0.01	1.16 ± 0.02	1.13 ± 0.02
The small intestine	3.35 ± 0.14	2.92 ± 0.20	3.01 ± 0.16	2.92 ± 0.20
The large intestine	0.602 ± 0.035	0.490 ± 0.035	0.645 ± 0.046 *	0.618 ± 0.045
Lengths of the large intestine (cm)	7.70 ± 0.20	7.30 ± 0.42	7.68 ± 0.18	8.18 ± 0.19
White adipose tissue				
Mesenteric	0.954 ± 0.089	1.22 ± 0.09	1.12 ± 0.16	1.05 ± 0.18
Perinephric	0.791 ± 0.097	0.981 ± 0.084	0.717 ± 0.092	0.623 ± 0.126 *
Epididymal	1.93 ± 0.13	2.63 ± 0.14	2.61 ± 0.24	2.38 ± 0.29
Total	3.67 ± 0.28	4.83 ± 0.22	4.45 ± 0.47	4.05 ± 0.59

Mean ± standard error, *n* = 7–8. CO: control diet, HF: high fat diet, ML: mushroom low (0.5%) diet, MH: mushroom high (3%) diet. ^†^: *p* < 0.05 vs. CO, *: *p* < 0.05 vs. HF.

**Table 6:** Biochemical parameters of serum and liver.

	CO	HF	ML	MH
Serum				
TC (mmol/L)	3.51 ± 0.13 *	4.23 ± 0.14 ^†^	4.97 ± 0.12 ^†,^*	4.25 ± 0.17 ^†^
TG (mmol/L)	2.39 ± 0.18	2.31 ± 0.10	2.35 ± 0.08	2.77 ± 0.13
PL (mmol/L)	17.1 ± 0.4	18.6 ± 0.6	21.1 ± 0.2 ^†,^*	19.4 ± 0.5 ^†^
TBARS (µmol/L)	7.37 ± 0.38 *	5.02 ± 0.28 ^†^	6.19 ± 0.26 ^†,^*	5.88 ± 0.08 ^†^
Glucose (mmol/L)	12.0 ± 1.1	13.1 ± 1.5	12.7 ± 0.5	9.94 ± 0.4
Insulin (ng/mL)	0.0747 ± 0.029	0.0777 ± 0.0275	0.231 ± 0.087	0.0684 ± 0.0223
ALT (IU/L)	7.20 ± 1.33	6.76 ± 0.65	7.79 ± 0.43	8.14 ± 1.39
AST (IU/L)	62.4 ± 5.6	66.7 ± 3.1	56.0 ± 5.1	49.6 ± 5.1
IL-6 (pg/mL)	29.2 ± 8.3	23.2 ± 8.9	39.6 ± 12.9	30.1 ± 8.4
Adiponectin (µg/mL)	33.6 ± 3.7	34.3 ± 3.5	32.3 ± 3.3	50.4 ± 3.6 ^†,^*
Liver				
TC (mmol/L)	1.82 ± 0.06 *	2.17 ± 0.09 ^†^	2.45 ± 0.06 ^†,^*	2.15 ± 0.05 ^†^
TG (mmol/L)	30.0 ± 6.84	36.9 ± 4.9	58.7 ± 7.6 ^†^	66.2 ± 7.4 ^†,^*
PL (mmol/L)	50.6 ± 2.6	50.6 ± 2.0	53.8 ± 1.3	50.1 ± 0.8
TBARS (µmol/L)	66.1 ± 2.7 *	50.7 ± 2.4 ^†^	59.8 ± 2.7 *	56.0 ± 2.2 ^†^

Mean ± standard error, *n* = 7–8, CO: control diet, HF: high fat diet, ML: mushroom low (0.5%) diet, MH: mushroom high (3%) diet. ^†^: *p* < 0.05 (vs. CO), *: *p* < 0.05 (vs. HF). TC: total cholesterol, TG: triacylglycerol, PL: phospholipid, TBARS: thiobarbituric acid active substances, ALT: alanine aminotransferase, AST: aspartate aminotransferase, IL-6: interleukin 6.

**Table 7:** mRNA expression levels in perinephric adipose tissue.

	CO	HF	ML	MH
*Fas*	1.00 ± 0.20	1.06 ± 0.07	0.970 ± 0.117	0.782 ± 0.126
*Me*	1.00 ± 0.18 *	0.526 ± 0.055 ^†^	0.396 ± 0.032 ^†^	0.617 ± 0.162
*G6pdx*	1.00 ± 0.16	0.678 ± 0.052	1.28 ± 0.22	2.27 ± 0.60 ^†,^*
*Srebp-1c*	1.00 ± 0.14 *	2.26 ± 0.22 ^†^	1.70 ± 0.13 ^†^	1.46 ± 0.28 *
*Hsl*	1.00 ± 0.20	1.05 ± 0.29	2.00 ± 0.52	3.13 ± 1.27 ^†,^*
*Pparγ*	1.00 ± 0.79	3.91 ± 1.90	2.61 ± 1.35	6.34 ± 1.61 ^†^
*Adipoq*	1.00 ± 0.23	3.71 ± 0.87	4.10 ± 1.48	9.31 ± 1.64 ^†,^*

Mean ± standard error, *n* = 7–8. CO: control diet, HF: high fat diet, ML: mushroom low (0.5%) diet, MH: mushroom high (3%) diet. ^†^: *p* < 0.05 (vs. CO), *: *p* < 0.05 (vs. HF).

## Supplementary Materials

The following are available online at http://www.mdpi.com/2072-6643/10/5/610/s1, Table S1: The relative abundance of gut bacterial phyla in mice. Table S2: The relative abundance of gut bacterial genera in mice.
