# Supplementary material for: Effects of Dietary Intake of Japanese Mushrooms on Visceral Fat Accumulation and Gut Microbiota in Mice
Source: Nutrients. 2018 May 14;10(5):610. doi: 10.3390/nu10050610 (PMC5986490; doi:10.3390/nu10050610)
Supplement: Supplementary file 1 [file nutrients-10-00610-s001.pdf]

nutrients

Supplementary: Effects of Dietary Intake of Japanese Mushrooms on Visceral Fat Accumulation and Gut Microbiota in Mice.

Takamitsu Shimizu[1*], Koichiro Mori[1], Kenji Ouchi[1], Mamoru Kushida[2] and Tsuyoshi Tsuduki[2]

Supplementary table legends

Table S1. The relative abundance of gut bacterial phyla in mice.
Table S2. The relative abundance of gut bacterial genera in mice.

Table S1 The relative abundance of gut bacterial phyla in mice.

	0 week	2 weeks				4 weeks			
Phylum		CO	HF	ML	MH	CO	HF	ML	MH
Bacteroidetes	0.393	0.299	0.182	0.152	0.209	0.484	0.370	0.132	0.190
Firmicutes	0.574	0.430	0.687	0.716	0.683	0.298	0.478	0.744	0.706
Proteobacteria	0.0118	0.0129	0.0609	0.0682	0.0611	0.0301	0.115	0.0450	0.0427
Actinobacteria	0.0131	0.257	0.0532	0.0488	0.0323	0.166	0.0211	0.0598	0.0570
Deferribacteres	0.00686	0.000838	0.00847	0.0120	0.0140	0.00186	0.0130	0.0192	0.00404
TM7	0.0000448	0	0	0	0	0	0	0	0
Tenericutes	0.000896	0.000279	0	0	0	0.000432	0	0	0
Verrucomicrobia	0	0.000124	0.00847	0.00300	0.000121	0.0203	0.00225	0.0000227	0.0000301
Other	0.000672	0	0.0000599	0.0000527	0.000121	0	0.0000775	0.000114	0.000121

Table S2 The relative abundance of gut bacterial genera in mice.

	0 week	2 weeks				4 weeks			
Genus		CO	HF	ML	MH	CO	HF	ML	MH
Bifidobacterium	0.000179	0.248	0.0393	0.0374	0.0194	0.153	0.0159	0.0447	0.0382
Adlercreutzia	0.0121	0.00276	0.0137	0.0106	0.0126	0.000919	0.00523	0.00800	0.0178
Bacteroides	0.0300	0.0785	0.0621	0.0491	0.0669	0.0852	0.132	0.0448	0.0582
Prevotella	0.0283	0.0405	0.0131	0.0113	0.00478	0.0977	0.0331	0.0134	0.00817
Mucispirillum	0.00686	0.000838	0.00847	0.0120	0.0140	0.00186	0.0130	0.0193	0.00404
Lactobacillus	0.0196	0.109	0.00485	0.00300	0.00187	0.0746	0.000906	0.00202	0.0366
Lactococcus	0	0.00552	0.0239	0.0209	0.0156	0.00157	0.00780	0.0190	0.04238
Streptococcus	0.0000896	0.0000310	0.000239	0.000290	0.0000605	0.0000270	0.000129	0.000227	0.000271
Dorea	0.0000896	0.000124	0.00168	0.000738	0.000272	0.0000270	0.00106	0.000977	0.000271
Roseburia	0.000224	0.000155	0.00856	0.00195	0.000302	0.0000540	0.00168	0.00309	0.000422
Ruminococcus	0.0126	0.00397	0.0245	0.0263	0.0304	0.00268	0.0106	0.0198	0.0267
Anaerotruncus	0.00148	0.000714	0.00470	0.00237	0.00305	0.000946	0.00479	0.00200	0.00133
Oscillospira	0.0345	0.0118	0.0587	0.0545	0.0408	0.0130	0.0558	0.0378	0.0269
Allobaculum	0.0000896	0.0623	0.192	0.216	0.174	0.0372	0.0467	0.335	0.219
Sutterella	0.00412	0.000403	0.000629	0.000580	0.00121	0.00268	0.000699	0.000182	0.00157
Escherichia	0.000134	0.0000931	0.0000299	0.000290	0.000181	0.000297	0.000337	0.0000455	0.000151
Akkermansia	0	0.000124	0.00847	0.00300	0.000121	0.0203	0.00225	0.0000227	0.0000301
